# Testing Concrete Sewer Maintenance Holes Using an Angular Modulated Penetrometer

**DOI:** 10.3390/ma17246187

**Published:** 2024-12-18

**Authors:** Sampath Thamel, Robert Ross, Alex Stumpf, Fernando Galetto, Jason Cotton

**Affiliations:** 1 Department of Engineering, La Trobe University, Bundoora, VIC 3086, Australia; s.warnakulasuriya@latrobe.edu.au (S.T.); a.stumpf@latrobe.edu.au (A.S.); f.galetto@latrobe.edu.au (F.G.); 2 Intelligent Water Networks, Melbourne, VIC 3000, Australia; jason.cotton@iwn.org.au

**Keywords:** wastewater infrastructure, infrastructure condition assessment, penetration testing, concrete testing, remote sensing, non-destructive testing

## Abstract

Around the world, a significant proportion of sewers and sewer maintenance holes are constructed from concrete. Unfortunately, one major problem with concrete sewer infrastructure is corrosion caused by biogenic hydrogen sulphide, which causes major issues for concrete structural integrity. Furthermore, concrete may be significantly corroded and softened but still pass a visual inspection. The novel system presented in this paper uses a penetrometer mounted on a robotic platform to measure the depth of penetration through a corroded concrete surface. An angular mechanism is used to rotate the penetrometer to new positions as striking aggregate may result in false readings. Based on laboratory analysis, this design is capable of providing consistent and precise multiple observations for both smooth and rough surfaces, as well as for flat and curved surfaces, with 0.1 mm accuracy. The use of a remote robotic platform eliminates the hazards of confined space entry whilst providing a repeatable analysis platform.

## 1. Introduction

Water and wastewater systems have had an enormous impact on public health, particularly over the last few hundred years [1,2]. In terms of water supply, some ancient Roman aqueducts are still in use today, although most drinking water is now conveyed through a modern network of pressurised pipes (which are not expected to have a service life of thousands of years) [3]. Historically, sewerage was buried, stored in cesspits, or dumped (e.g., into waterways) [1,4]. We now know that the safe management of wastewater sewerage is crucial for maintaining healthy ecosystems, preventing disease and contamination. According to the 2021 National State of the Assets Report, Water & Wastewater infrastructure assets in Australia have a staggering combined value of AUD 62 billion, with an estimated replacement cost of AUD 99 billion [5,6]. Australia’s sewerage infrastructure comprises an extensive network of pipes spanning over 110,000 km, with an aggregate value of AUD 35 billion [7]. Wastewater sewers play a vital role in transporting used water from various sources, such as sinks, toilets, showers, and industrial processes, to treatment plants where it is purified before being discharged into oceans, rivers, or lakes, or reused for non-potable purposes.

The consequences of failure within the sewer network are significant, affecting public health, environment, and economic stability [8]. Such failures can result in the discharge of untreated wastewater, causing property damage and exposing the community to harmful pathogens, bacteria, and viruses [9]. This, in turn, can lead to the spread of waterborne diseases such as cholera, typhoid fever, and gastroenteritis, posing a substantial risk to public health whilst contaminating surface water bodies such as rivers, lakes, and streams, along with groundwater sources. Contamination can compromise drinking water quality and harm aquatic ecosystems and wildlife.

Sewer networks, both old and modern, have been constructed using a variety of materials, including bricks, Vitrified Clay (VC), PolyVinyl Chloride (PVC), Asbestos-Cement (AC), and concrete [10]. Although concrete maintains a long service life for general use and storm-water pipes, the sewer network presents a unique challenge. Concrete assets within the sewer network are susceptible to Microbiologically Induced Corrosion (MIC), which occurs due to biogentic hydrogen sulphide (H_2_S) attack produced by Acidithiobacillus thiooxidans sulphide-oxidising micro-organisms [11,12]. This rate of corrosion varies widely with temperature, concrete composition, microorganisms present, pH, location, and localised H_2_S concentration [11,13]. As the concrete is corroded, it transforms into a layer of non-structural gypsum. Studies have shown that MIC can decrease the expected lifespan from 100 years to 30–50 years, and in extreme cases it can even reduce it to 10 years or fewer [14,15,16]. Understanding and addressing MIC is crucial for the long-term durability and performance of concrete sewer systems.

A major challenge with MIC in sewer pipes is that it can often go undetected during visual inspection, such as when using Closed-Circuit Television (CCTV) [17,18]. Although the outer layer of concrete may have significantly softened and is on the verge of breaking away, it can appear relatively intact. Once this layer detaches, it reduces the protective cover over the rebar, increasing the surface area for concrete carbonisation and heightening the risk of rebar corrosion, leading to further cracking and failure of the structure.

Modern sewer networks comprise a variety of different assets, including pipes, pump stations, wastewater treatment plants, and maintenance holes (formerly called manholes). Sewer maintenance holes are commonly constructed as pre-fabricated concrete structures and constitute an essential part of the infrastructure that ensures the efficient management and maintenance of underground sewer systems. Maintenance holes are strategically placed at intervals of 50 to 200 m throughout the national sewer infrastructure to provide access for maintenance, inspection, and emergency response.

These maintenance holes are not only essential for sewer workers but also serve as the only point of interaction between the general public and the sewer network, as they are often stepped on by pedestrians. Despite their importance, the current condition of these assets is not well-documented, posing a significant risk to both water utilities and public safety. Multiple potential failure modes (e.g., cracking, MIC) further exacerbate the risks associated with the condition of these critical sewer system components. Working around maintenance holes, along with their associated sewers, poses several significant hazards, including potentially dangerous and explosive gases, biological risks, chemical hazards, falls from heights, structural integrity risks, and drowning hazards [19]. Due to the perilous nature of this environment, any work carried out within these chambers necessitates confined space entry procedures and comprehensive risk assessments [20].

Limited qualitative information is available regarding the structural condition of these sewer maintenance holes. The current inspection procedures, which primarily consist of visual inspection or core drill sampling, have respectively proven to be inadequate or not cost-effective for widespread use [21]. This limitation poses a significant risk to public safety and service personnel, as these maintenance holes are present in every Australian neighbourhood. In response, this project aims to develop and implement a practical, low-cost solution that can effectively assess the condition of concrete sewer maintenance holes and enable timely remediation and prioritisation.

A visual inspection does not provide an accurate determination of concrete corrosion. The relative softness of the corroded concrete (it can crumble when gently probed) offers a direct tactile methodology for testing concrete corrosion. Corrosion could either be cleaned off (e.g., brushed or pressure washed) and analysed in terms of how much aggregate is exposed or probed using a penetrometer to determine how much of the outer layer of the concrete is degraded [22,23]. While both methods affect the surface of the concrete, they can generally be classified as Non-Destructive Testing (NDT) since the surface layer does not contribute to the overall structural integrity (cleaning before visual inspections is relatively common). Excessive removal of this outer layer may lead to more rapid corrosion, but more research in required into the level of protection provided by this non-structural layer [24]. A challenge with this probing method is that it produces data that can vary significantly based on the position of the aggregate at the penetrometer contacts. Therefore, it is necessary to conduct tests at multiple locations.

Two testing methods are currently employed to evaluate the softness of corroded concrete: the penetrometer test and the drill-resistance test. The drill resistance test primarily serves as an indirect method for estimating the strength of concrete. Nevertheless, it can also be utilized to measure the penetration of corroded concrete layers. By establishing a correlation between the drilling resistance values and the concrete’s compressive strength, it becomes possible to estimate the concrete’s strength. Based on drilling time, sound levels, and impact sound, the quality of concrete has been characterised for several types of concrete surface [25,26]. Nevertheless, in addition to assessing the concrete’s strength, drilling resistance was utilized to evaluate the extent of corroded concrete [27]. However, during the drill-resistance test, there is an increased likelihood of damaging the concrete, as it can easily penetrate the harder surface through the corroded layer and expose the rebar. This poses a significant problem, as it could accelerate the corrosion of the rebar. Therefore, drill-resistance is not commonly used to assess the penetration of corroded concrete layers.

In comparison to the drill-resistance method, the penetrometer is a widely accepted approach for assessing the depth of the corroded concrete layer, as it interacts only with the softer layers. The penetrometer test uses a steel probe with a blunt conical tip that is driven to penetrate through concrete to a certain depth or a prescribed force threshold [23]. This test can act as a preliminary or indicative measure for evaluating concrete. It offers a quick and convenient method to gauge concrete’s surface hardness and potential uniformity. However, to achieve a thorough evaluation of concrete properties, other testing methods, like core sampling or various non-destructive techniques, may be required.

The penetrometer test has significant implications, not only for concrete but also for various tests on other building materials. A detailed discussion has been provided on the compressive strength of different types of mortars in masonry joints, based on how far the needle probe of the RSM-15 penetrometer penetrates [28,29]. Alongside the analysis of compressive strength, the penetrometer is also capable of evaluating the mechanical properties of mortars in masonry joints [30]. To facilitate a fast and easy method for the penetrometer test, a new idea was proposed involving a steel spring with a specific stiffness akin to that found in air-soft guns [31]. This design allows for quicker assessments and reduces the influence of human error on the final results. Alongside the probe penetration technique utilized in the penetrometer test, the miniature ball penetrometer method was employed to assess the shear strength of the testing samples [32]. Penetrometer testing can quantify layers of degraded concrete that are no longer structurally sound, but results can be inaccurate depending on the test location (for instance, if the probe encounters aggregate). Additionally, the penetrometer test can be used alongside pH sensors to obtain more reliable data, reflecting the hardness and extent of damage in deteriorated concrete surfaces. Thus, it can help to clearly detect and locate the actual degradation caused by Microbiologically Induced Corrosion (MIC) because it causes significant differences in the pH measurement. A research study indicated that a pH depth profile can illustrate the diffusion, formation, and chemical reaction of sulphuric acid within concrete [33].

One of the primary challenges associated with conducting penetrometer tests in sewer maintenance holes is the need for confined space entry. In addition to this challenge, obtaining multiple readings can result in various difficulties and limitations, particularly in deep maintenance holes. As highlighted in the literature review, most available penetrometers are designed for operation by human operators. This poses significant challenges and limitations for assessing the condition of concrete in sewer maintenance holes, as the operator must navigate confined spaces. To address this issue, this study introduces a novel penetrometer testing method utilising an Angular Modulated Penetrometer Testing (AMPT) robot, enabling remote execution of the penetrometer test.

The fundamental contribution of this paper relates to the design, development, and evaluation of a purpose-built robot remote sensing platform—the Angular Modulated Penetrometer Testing (AMPT) robot. A key objective of this research study is to confirm the repeatability and precision of multiple observations during laboratory testing across a variety of surfaces, including both smooth and rough, as well as flat and curved, surfaces. Also, through various observations, the maximum penetration in specific areas can be accurately calculated with great precision. Additionally, the stability of the Sensor Deployment Mechanism is also assessed through laboratory testing. The AMPT Robot is an adaptable remote sensing platform designed to deploy a penetrometer within concrete maintenance holes in a way that avoids confined space entry. For the penetrometer, a custom-designed system comprising a load cell, a stepper motor, thrust bearings, gear wheels, and a linear actuator allows the precise position of the probe to be adjusted to automatically perform multiple measurements at different locations. This robotic design ensures that the testing can be performed in a safe and repeatable manner. Additionally, the AMPT Robot features adjustable components, enabling it to modify its size according to the specific testing environment. This versatility allows it to perform penetrometer testing across a broad range of commonly used sewer maintenance holes throughout Australia.

The remainder of this paper is organised as follows. Section 2 discusses the design and implementation of the robotic probing system. Section 3 describes the laboratory experimental study. Section 4 presents and evaluates the results of the experimental work. Finally, concluding remarks and future work are presented in Section 5.

## 2. System Design

Obtaining accurate readings using a traditional penetrometer test is challenging, particularly because the penetrometer tip could hit coarse aggregates. This problem is especially pronounced in highly degraded sewer maintenance holes, where achieving consistent and reliable measurements is difficult. To address this issue, this study proposes an innovative robotic solution, the AMPT Robot. This design allows the penetrometer to take multiple readings at different locations near the initial test point using the same tip configuration. By comparing these multiple readings, the maximum penetration depth can be validated, significantly reducing the risk of inaccuracies caused by contact with aggregates. The AMPT Robot consists of two main components: the Sensor Deployment Mechanism and the Penetrometer Mechanism.

### 2.1. Sensor Deployment Mechanism

The Sensor Deployment Mechanism (SDM) is engineered to meet all critical requirements for deploying penetrometer testing within sewer maintenance holes. Accurate positioning of the probe is crucial for penetrometer testing. Thus, the primary role of the SDM is to uphold a perpendicular arrangement, offering a stable base for precise measurement. Moreover, maintenance holes can vary greatly in diameter, usually ranging from 600 to 1100 mm, which is why the SDM features adaptable characteristics to fit this spectrum. This adaptability is achieved through two approaches: interchangeable mechanisms designed for specific diameters and adjustable components. Achieving rigidity once the mechanism is extended to provide stability within the maintenance hole is another key requirement addressed by the SDM. Any malfunction in the mechanical constraints could result in significant complications during testing. Additionally, the SDM is engineered to reduce damage to the surface during operation. As many maintenance holes possess corroded or weakened concrete surfaces, the SDM incorporates features that distribute the force at the contact points, thereby decreasing the likelihood of additional surface damage during deployment.

**Figure 1:**
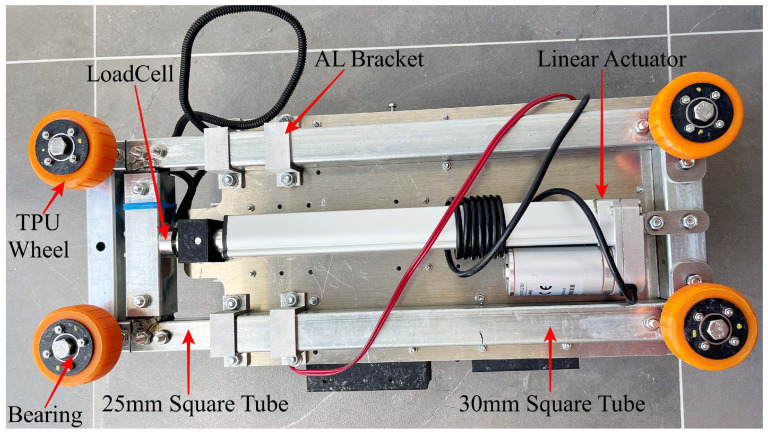
Basic elements of the AMPT Robot (bottom view).

A simplified SDM design, shown in Figure 1, addresses key requirements such as technical limitations, material accessibility, design complexity, size, and weight, with a primary focus on length. The SDM is designed to fit maintenance holes with diameters between 600 and 1100 mm. It can compress to 495 mm for a 600 mm maintenance hole and extend to 1150 mm for larger ones, ensuring collision-free operation. The design uses a telescopic arrangement of galvanised steel Rectangular Hollow Section (RHS) in three sizes (20 mm, 25 mm, and 30 mm), providing a practical and efficient solution for penetrometer testing.

**Figure 2:**
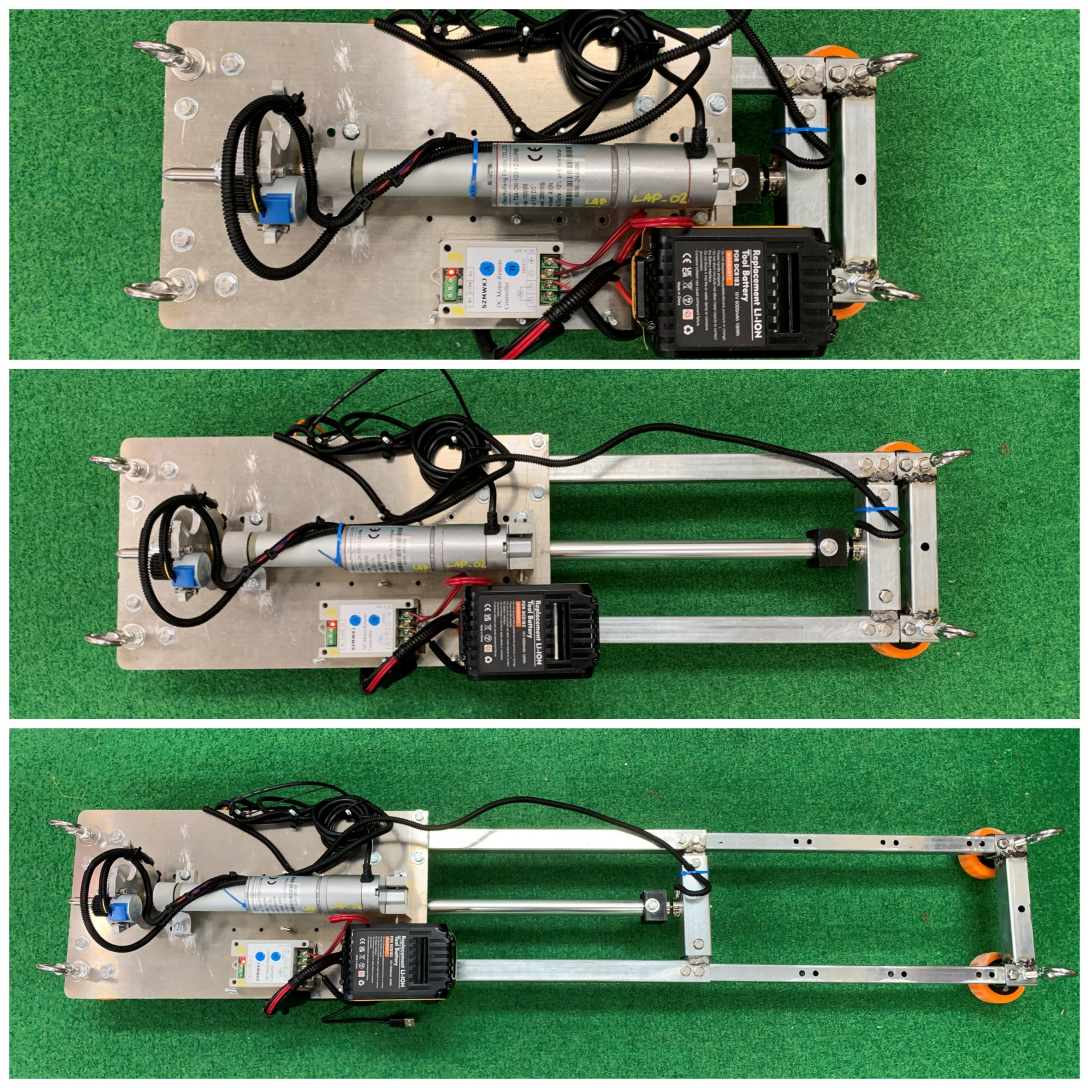
Sensor Deployment Mechanism extension.

The SDM can be adjusted to predefined length increments based on the specific testing location, as shown in Figure 2. These adjustments are achieved in 100 mm increments by extending or retracting the 20 mm RHS—providing a convenient and flexible control over the SDM’s overall size.

The main platform of the SDM is made of 6mm aluminium alloy plate. The 30 mm RHS steel tube is mechanically fastened to the aluminium plate, as shown in Figure 1. A telescopic arrangement is comprised as segments of 25 mm RHS slides within the 30 mm RHS and segments of 20 mm RHS slide within the 25 mm RHS. This telescopic arrangement (shown compressed in Figure 1) allows for controlled extension and retraction for deployment and retrieval within the maintenance holes.

The telescopic arms are extended using an electro-mechanical linear actuator that has a force rating of 1000 N and a stroke length of 200 mm, which is suitable considering the design’s weight and operational needs. A miniature compression load cell with a rated load capacity of 300 kg is attached between the stroke of the linear actuator and the square tube through a 3D-printed bracket. The purpose of this load cell is to monitors and control the force exerted by the wheels on the surface to minimise any potential damage.

The SDM mechanism incorporates four wheels, as shown in Figure 1. The wheels are composed of three parts: an orange section made from Thermoplastic Polyurethane (TPU) for flexibility, a black section made from Polyethylene Terephthalate Glycol (PETG) for strength, and two deep groove bearings to support smooth rotation. This design holds the SDM in place and maintains acceptable pressure as it deforms to the shape of the maintenance hole wall. Each wheel is attached to the SDM via a single bolt, which allows it to be rotated around the inside of the maintenance hole when only a moderate force (e.g., 20 N) is used.

**Figure 3:**
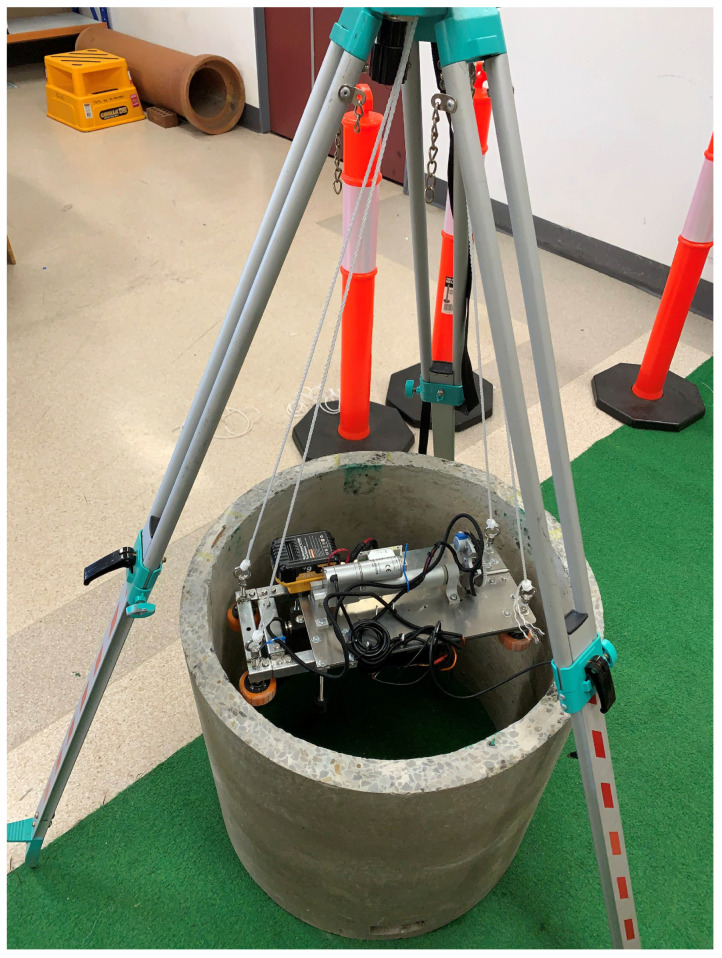
Tripod-based rope arrangement for AMPT Robot.

For deploying the AMPT Robot safely into a maintenance hole, a tripod-based rope arrangement is proposed, as shown in Figure 3. This setup uses four ropes for controlled lowering and rotation of the SDM from the surface level. This allows the SDM to be positioned remotely, which improves the safe operating of the device as a human operator is not required to enter the sewer maintenance hole.

### 2.2. Penetrometer Mechanism

**Figure 4:**
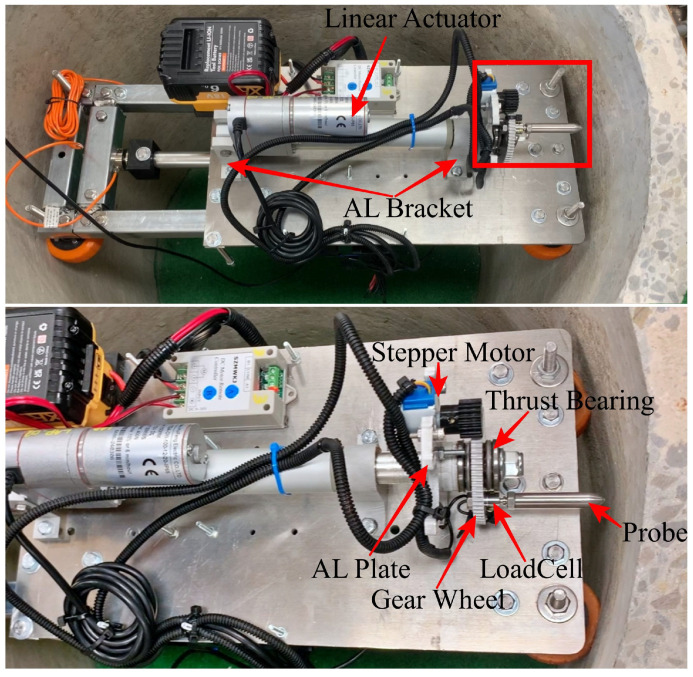
Basic elements of the AMPT Robot (top view).

**Figure 5:**
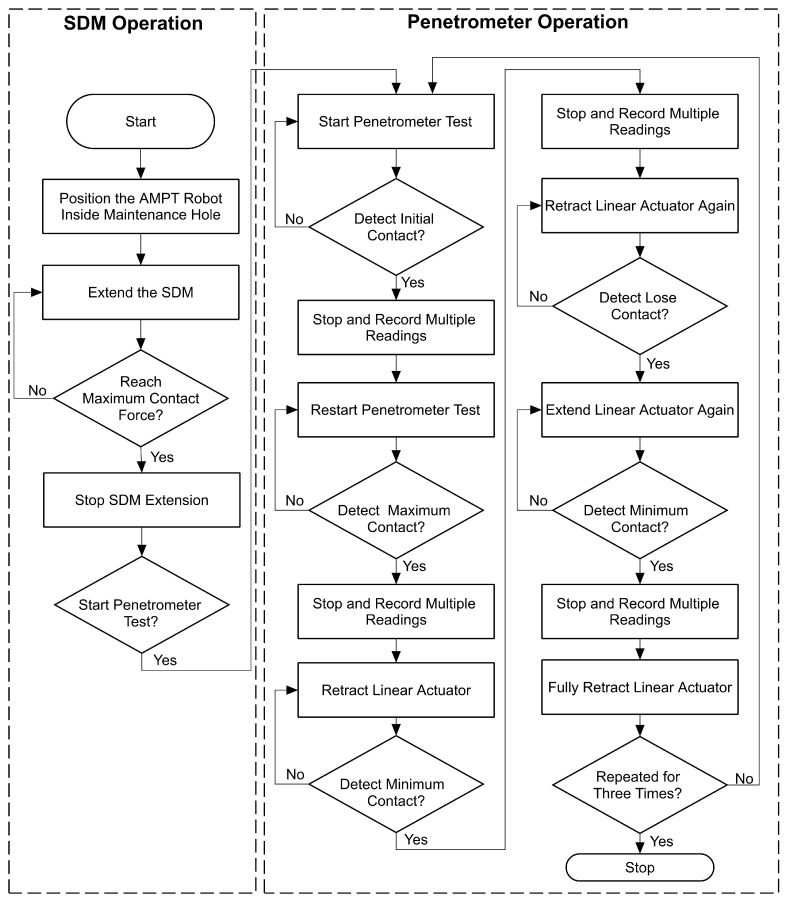
Flowchart of system operation.

The penetrometer sensor system comprises a linear actuator, stepper motor, gear wheel, load cell, and probe, as illustrated in Figure 4. The purpose of the penetrometer is to probe into the outer surface of the maintenance hole, thereby quantifying, in terms of thickness, the amount on non-structural degraded concrete which is sitting on top of the structural concrete. The linear actuator extends the probe forward and backwards whilst the force on the probe is measured with the load cell (see Figure 5).

Through experimentally testing various designs, it was established that the maximum force applied to the surface should be 200 N. This force is adequate to penetrate corroded concrete but is not strong enough to damage structural concrete. To meet this requirement, a customised electro-mechanical linear actuator with a load capacity of 400 N and speed of 20 mm/s was used with feedback to determine the stroke displacement with an accuracy of 0.1 mm.

The linear actuator serves as the core element for driving and measuring the penetration of the probe. The linear actuator is positioned close to the base of the SDM to optimise the distribution of reaction forces along the wheels. Connected to the linear actuator is a probe system comprising a threaded rod, two thrust bearings, a gear wheel, and a nylon lock nut.

A small stepper motor is used to rotate the main gear that the probe is attached to and is coupled using a simple spur gear. This rotation allows the probe to be used to test multiple locations without moving the SDM. A micro-load cell was used due to its convenient attachment method to the gear wheel, size, and load capacity. The design of the penetrometer tip (Extruded round tip) was chosen based on it demonstrating the least slippage when used for concrete condition assessment [22]. In this design, the opposite end of the probe is drilled and tapped for an M6 thread, allowing for easy attachment to the M6 thread of the load cell, as shown in Figure 4. This feature offers a more convenient method for connecting various probe designs utilized in earlier research to the same penetrometer mechanism. This will also facilitate the execution of multiple studies on different probe design types. A thrust bearing was secured to the rod using a nut to complete the mechanism. The thrust bearing is crucial in reducing friction on the gear wheel, ensuring smoother operation. Additionally, it offers a convenient solution for securing all parts within the stoke without undesirable play in the mechanism. This configuration allows the gear wheel to be smoothly rotated to the desired position by the stepper motor.

### 2.3. Controller Design

**Figure 6:**
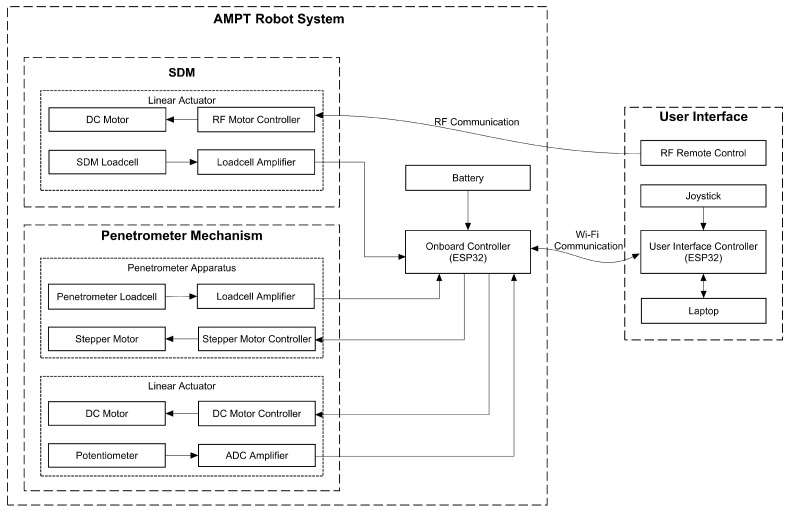
Block diagram of system design.

The testing process entails managing two primary components: the linear actuator, comprising a penetrometer, and a SDM extension linear actuator, as detailed in the block diagram in Figure 6. Wireless communication (ESP-NOW protocol) is used for controlling the AMPT Robot, which facilitates fast data communication and avoids the possibility of snags or tangled wires.

**Figure 7:**
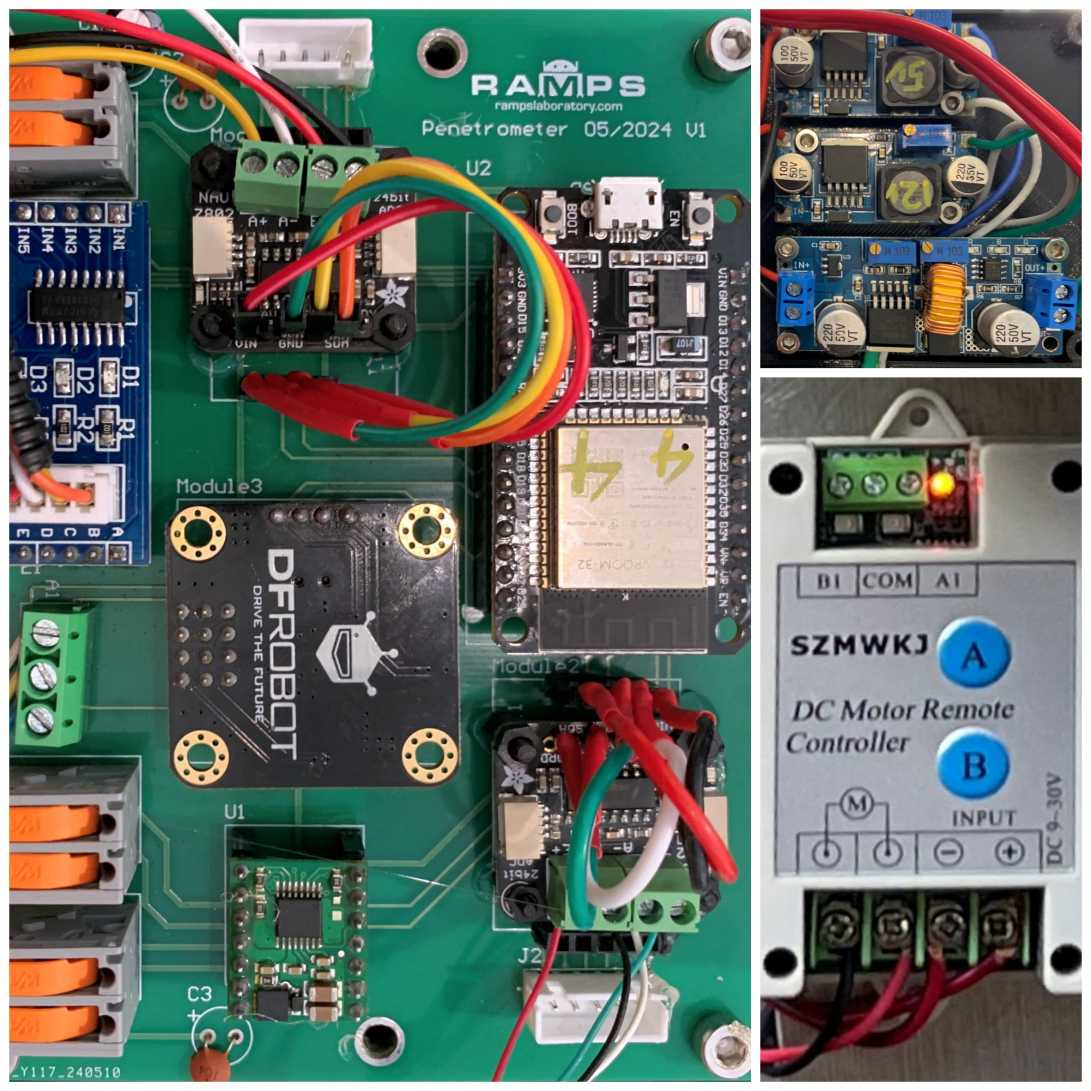
Electrical subsystem design for the controller unit of AMPT Robot.

An ESP32 was selected for speed, memory capacity, and built-in Wi-Fi connectivity. For controlling the SDM extension linear actuator, a DC motor remote-control module is used, as illustrated in Figure 7, as this provides a more convenient approach for the user who may wish to visually observe the robot while it is extending out. Commands are sent using a separate RF remote control. The load cell reading can provide insights into the force exerted on the surface and how these change as the probe is extended. The connection between the load cell amplifier and the ESP32 is through I^2^C, enabling real-time data transmission to the user interface device.

When it comes to controlling the penetrometer operation, the user gives commands to the AMPT Robot through the laptop user interface. Data are received by the laptop and stored directly to an Excel spreadsheet. A portable lithium-ion drill battery is used to power the robot and enables rapid field swap-over when flat. To supply power to the linear actuators and control board, 12 V and 5 V step-down voltage converters are employed, as illustrated in Figure 7. The penetrometer mechanism includes a stepper motor and a load cell. The stepper motor rotates the gear wheel to position the penetrometer based on a preset location. In this project, a 28BYJ-48 stepper motor is used due to its size, voltage rating, and torque capacity. Communication between the load cells is established using I^2^C communication through the NAU7802 load cell amplifier (Nuvoton, Hsinchu, Taiwan).

### 2.4. Observation of Penetrometer Displacement

Diverse penetrometer designs are currently utilised in various research and industrial settings, tailored to the specific applications and types of data being observed [28]. Despite these variations, the underlying principle remains consistent across all designs—an initial force is exerted at the testing location, resulting in the creation of penetration through the surface. Subsequently, the depth of penetration is measured using precise tools such as a Vernier caliper, ensuring the attainment of accurate and consistent observations. ASTM C803/C803M elaborates on two distinct penetration resistance methods: resistance testing with probes and resistance testing with pins [34]. In both testing methodologies, the depth of penetration is measured immediately after the load is removed from the probe or pin, ensuring a precise assessment of the penetration depth.

Measuring penetration under a load presents several challenges in terms of accuracy and repeatability. The robot design comprises the SDM and the penetrometer mechanism. When measuring penetration under a load, issues arise due to compliance in both mechanisms. For instance, the SDM incorporates semi-flexible wheels (compressed against the wall surface), which can be compressed further when probing with the penetrometer. Furthermore, the distance between the penetrometer tip and the front wheel varies by approximately 135 to 85 mm, resulting in inconsistent compliance based on the tip’s location. Additionally, there may be other concealed mechanical constraints to consider. During laboratory testing, the total compliance was quantified using a digital dial gauge, revealing variations of around 1 mm between when the probe was just touching the wall (0 N load) to when the maximum (200 N) load was reached.

To address these mechanism compliance limitations, the study has implemented a straightforward technique, as illustrated in Figure 5. The fundamental principle behind this technique is to measure the penetration with minimal force—thereby reducing effects due to mechanical compliance. As shown in Figure 5, once the probe makes initial contact with the surface, the linear actuator of the penetrometer mechanism is halted for up to 100 ms, and multiple readings are taken. Based on laboratory testing, the initial contact with modelling clay is defined as 0.4 N. Following this, the penetrometer mechanism is moved until it reaches the maximum-rated load, approximately 200 N. At this stage, the linear actuator is once again halted for around 100 ms. Subsequently, the linear actuator is retracted until it reaches less than the defined minimum contact force (40 N based on lab testing). Upon reaching less than the minimum contact force, the linear actuator operation is stopped. Once this is completed, the linear actuator is retracted until it loses contact with the clay. After this, the penetrometer mechanism is moved forward again until it reaches the minimum contact force defined earlier. Following this, the operation is stopped for around 100 ms (allowing multiple samples to be recorded). Once this step is completed, the penetrometer will return to its initial position.

The above techniques allow it to observe actual penetration in two cases: under load (first test) and with minimal load (subsequent tests). This occurs as the first test (with 200 N) permanently deforms the soft clay surface (except for a small amount of spring-back). Hence, a 40 N load point should be the same point minus any compliance found in the mechanism.

### 2.5. Operation

When preparing to conduct testing in a sewer maintenance hole, it is essential to customise the length of the SDM to accommodate the specific testing requirements. As previously discussed, the linear actuator utilised for the SDM allows for a maximum extension of 200 mm. Furthermore, a pre-set adjustment increment of 100 mm has been provided to give flexibility to adapt to diverse maintenance holes. The AMPT Robot can be carefully lowered into the maintenance hole using a system of ropes attached to a tripod. Once the robot is positioned at the testing location, the SDM is extended using the RF remote control. The extension process is managed by controlling the linear actuator, with guidance provided by load cell data.

**Figure 8:**
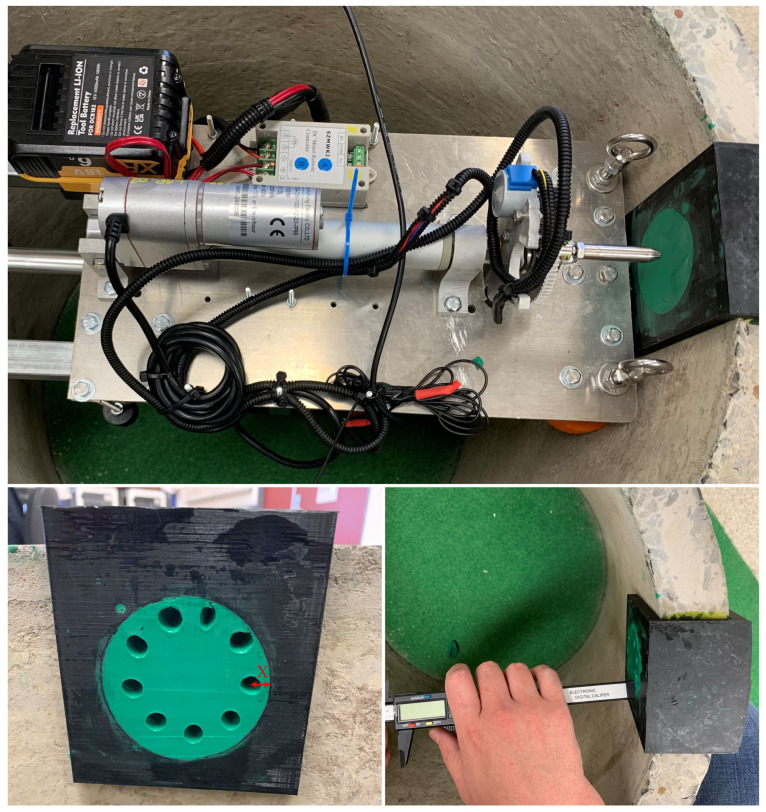
Experimental study of penetrometer test with a reinforced concrete sewer pipe.

With the SDM properly positioned, the testing process is initiated via a joystick command. After receiving the command, the linear actuator with the penetrometer probe moves forward until the probe makes initial contact with the surface. Upon contact with the surface, the controller monitors the stroke displacement as initial contact. Subsequently, the probe continues to advance until it reaches a maximum contact force of 200 N. Once the maximum force is reached, the linear actuator stops and returns to the initial position. If the probe penetrates more than 20 mm after the initial contact with the surface, the entire process halts, and the linear actuator returns to its initial position. This step will continue for up to three multiple readings on the same point. Upon completing the aforementioned steps, the controller rotates the gear wheel approximately 45 degrees clockwise and repeats the process. In this scenario, the gear wheel is controlled to reach 345 degrees, with the final position shifted by only 30 degrees. In this configuration, a complete 360° rotation is restricted by mechanical arrangements to enhance the design’s safety. Consequently, it offers a total of nine positions for observation, as illustrated in Figure 8. Upon completion of all the readings, the gear wheel is rotated counterclockwise by 345 degrees to return to the initial position.

During the entire operation, the control box transmits real-time data to the user interface. These data are presented visually on an Excel spreadsheet and include the displacement of the linear actuator stroke (in millimetres), load cell readings for penetration (in Newtons), and the contact force of the SDM (also in Newtons). The collected data can then be used to generate graphs, allowing for an effective observation of the maximum penetration achieved on a given surface.

## 3. Experimental Study

One of the primary challenges that motivated the development of an angular modulated penetrometer is the need to address the limitation of inaccurate observations when the penetrometer encounters aggregate. To overcome this limitation, the proposed solution uses multiple readings taken from different angles and positions to account for inconsistencies caused by aggregate. Furthermore, the repeatability of observations is crucial during field testing, especially considering the expectation of conducting multiple tests in specific locations. To further validate these requirements before field testing, this paper records laboratory tests using clay as an analogue for degraded concrete to test repeatability and accuracy for both a reinforced concrete sewer pipe and a concrete block with exposed aggregate.

### 3.1. Penetrometer Test with Reinforced Concrete Sewer Pipe

This study uses a reinforced concrete sewer pipe with an internal diameter of approximately 600 mm (DN600) and a thickness of around 50 mm. To begin, the AMPT Robot is positioned inside the sewer pipe and securely fixed by extending the SDM until it firmly contacts the wall, as depicted in Figure 8. Subsequently, the test specimen is affixed to the sewer pipe, and its position is adjusted based on the placement of the penetrometer. The test specimen comprises two parts: a 3D-printed plastic bracket and modelling clay (green colour). Based on previous studies, modelling clay is used to simulate the corroded concrete layer, specifically identified as the gypsum (CaSO_4_) layer [35]. This is to simulate Microbially Induced Corrosion (MIC) in sewer pipes, which results in the outer layer of concrete transforming into a softer layer. Consequently, this layer becomes so soft that it can be easily scratched with a fingernail. As a result, the hardness of the corroded concrete layer reaches a Mohs hardness of approximately 2 [36]. Given its softness, modelling clay exhibits similar behaviour, possessing a Mohs hardness of 1. This approach effectively replicates the softness characteristic of the gypsum layer.

The 3D-printed bracket is specifically designed to match the shape of the sewer pipe, offering a more convenient approach to securely fixing the pipe and adjusting its position. Moreover, the middle section of the 3D-printed plastic bracket, filled with modelling clay, is designed according to the operating area of the penetrometer. Hence, the minimum distance between the middle of the penetration hole and the adjacent 3D-printed plastic bracket was designed as 8 mm, as illustrated by the ‘X’ mark in Figure 8. Thus, it provides a close flat surface that serves as the reference plane when observing the actual penetration using the Vernier caliper, as illustrated in Figure 8. Before the experimental study, the stroke displacement was calibrated using a digital dial gauge.

Before starting the experimental study, the plastic bracket was affixed along the sewer pipe, ensuring it aligned with designated penetrometer testing sites. After completing these initial steps, the user issued a command to begin the first reading. The procedure for the penetrometer test, including repeatable observations, is depicted in Figure 5. Following this, the user instructs a rotation in the penetrometer’s position and repeats the same steps. This entire process is carried out for a maximum of 9 positions, resulting in a cumulative total of 27 observations for each SDM testing location. The observations from the 9 points are shown in Figure 8.

This experimental study involved conducting tests at 5 distinct locations and recording 135 observations on sewer pipes. After each test, the penetration depth was measured from the top clay surface to the sound concrete using a Vernier caliper, as shown in Figure 8. Prior to taking measurements with the Vernier caliper, the surface was flattened using a tool to ensure a uniform and reliable reference surface for accurate measurements. Additionally, to facilitate seamless penetration and prevent clay from adhering, a thin layer of cooking oil was applied to the penetrometer probe before each test. Due to the roughness of the surface, there were some difficulties when observing more reliable penetration with the Vernier caliper. As an effective solution, multiple readings were recorded and averaged.

### 3.2. Penetrometer Test with Concrete Block

**Figure 9:**
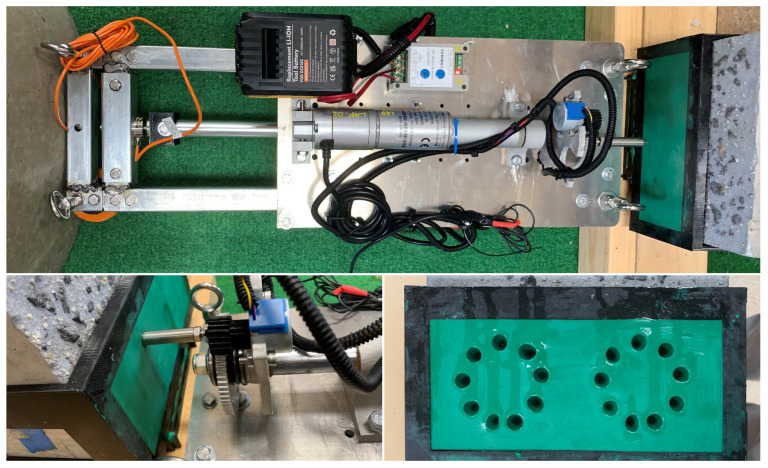
Experimental study of penetrometer test with concrete block.

In addition to the curved surface used earlier, a concrete block with a flat surface was used for lab testing. This concrete block contains 10 mm exposed aggregates, which match the recommended coarse aggregates for sewer maintenance holes according to AS 4198 standards [37]. The dimensions of the concrete block were approximately 200 mm × 200 mm × 100 mm. To examine the effect of exposed aggregates during penetrometer testing, a concrete surface retarder was utilised to uncover the coarse aggregates on the surface, with the goal of simulating the exposed aggregate found in a deteriorated sewer pipe. This was achieved by spraying a surface retarder on the concrete block’s surface after fabrication, which delayed the concrete’s setting time on the top surface. After 24 h, pressurised water was used to remove the cement and fine aggregates from the surface, resulting in a rough surface similar to what is encountered in a degraded sewer environment. This test specimen was water-cured for up to seven days before testing. Similarly, in this study, a 3D-printed plastic bracket was used, along with modelling clay to mimic the gypsum (CaSO_4_) layer. Once the 3D-printed plastic bracket was positioned on the concrete block, the void was filled with modelling clay and the surface was levelled. The testing robot was securely attached using separate attachments, as shown in Figure 9. After setting it up, the placement of the 3D-printed plastic bracket was adjusted based on the operating area of the penetrometer.

Following the same procedure used for reinforced concrete sewer pipe, data were recorded on an Excel 365 spreadsheet for two locations, as illustrated in Figure 9. A total of 52 observations were recorded. As before, after conducting penetrometer testing, the actual penetration was measured using a Vernier caliper. However, measuring the actual penetration had some limitations due to the presence of coarse aggregates. The configuration and alignment of the visible coarse aggregates complicated the accurate measurement of penetration with the Vernier caliper. This challenge arose because the depth measurement component of the Vernier caliper is significantly smaller than the cross-sectional area of the penetrometer probe, requiring it to be placed in several positions within the penetration hole created by the probe. To address this issue, several measurements were taken and then averaged.

### 3.3. Stability of Sensor Deployment Mechanism

In addition to analysing the penetrometer, the performance of the SDM was further investigated under a load. In this stage, a separate study was conducted to study the stability of the SDM. The presence of water and deposited sewerage contaminates inside the sewer maintenance hole wall and the absorption of water due to degradation can create slippery conditions. This may lead to difficulties in maintaining a stable arrangement during the penetrometer testing. Additionally, insufficient contact and traction between the wheel and the wall can pose limitations and challenges when conducting stable observations. These issues can be exacerbated in heavily degraded sewer maintenance holes. An experimental study was conducted in the laboratory to gain further insights into these behaviours.

In this experimental setup, a slippery surface on the wall was created by spraying water and water mixed with detergent. The experiment was repeated multiple times with varying amounts of detergent. A tripod mechanism secured the testing robot inside a reinforced concrete sewer pipe, as illustrated in Figure 3. Before the extension of the SDM within the sewer pipe, a level tool was used to confirm that the SDM was placed parallel to the ground. This was easily achieved by adjusting the length of the four ropes connected to the tripod. Once the AMPT Robot was in position, the SDM was extended to apply a pre-defined force range on the wall. The force applied on the wall was measured using a load cell attached to the SDM linear actuator. A digital luggage scale was then attached to ropes. After the setup was completed, two people attempted to lift the testing robot, and the minimum load required to move the robot from its initial location was recorded. Meanwhile, the change in contact force on the wall was monitored using the load cell readings, which were saved in an Excel spreadsheet throughout the experimental study.

## 4. Results and Evaluation

This section presents results for the test specimens of both reinforced concrete sewer pipes and concrete blocks. One of the main objectives of this study is to evaluate the accuracy of the readings, repeatability, and calculation of maximum penetration.

### 4.1. Validation of Penetrometer Test with Reinforced Concrete Sewer Pipe

**Figure 10:**
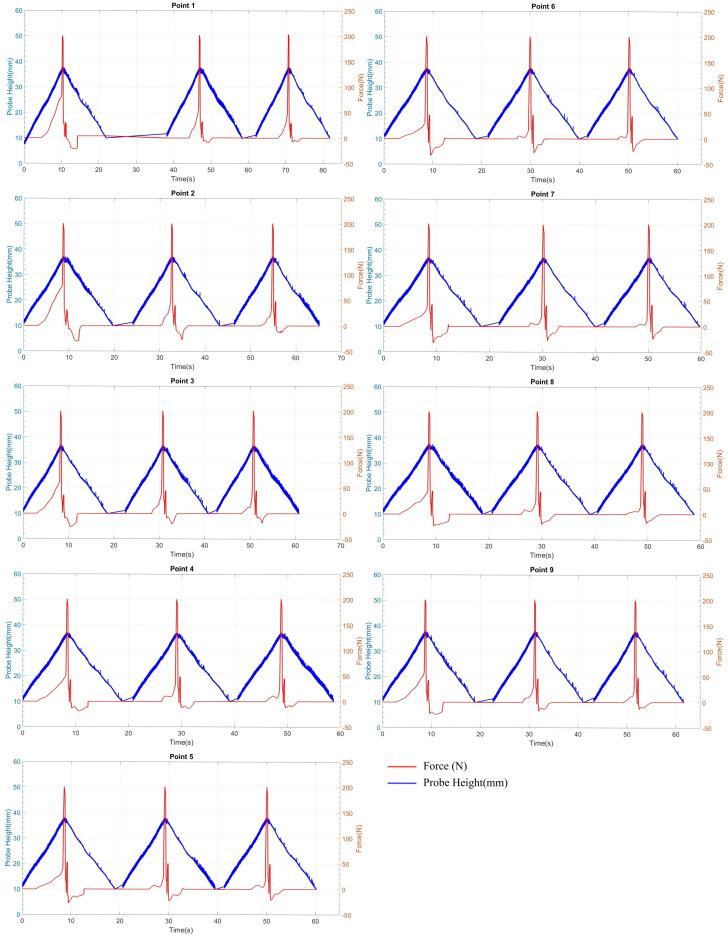
Penetrometer test with reinforced concrete sewer pipe and clay for nine test sites.

As detailed in Section 3.1, five sites were assessed, resulting in 135 recorded readings. This paper provides data for only one specific location as a representative sample as the other test locations yielded near-identical results. As illustrated in Figure 8, the testing was performed at a designated spot, with nine points evaluated through three repeated observations for each site. Therefore, a total of 27 observations are summarised, and for each observation approximately 16,000 sample points were recorded. According to Figure 10, it was evident that when the probe first contacted the surface, there was a gradual increase in force. This occurred as the probe penetrated the clay until it encountered a firm surface (concrete behind the clay). Based on the experimental findings, the minimum force necessary to penetrate the clay was approximately 20 N, though it varied slightly depending on the filling of the plastic bracket. Upon reaching the harder surface, there was a sudden surge in force, and the process was halted automatically when it reached 200 N. Subsequently, the penetrometer retracted until a contact force of approximately 2 N was achieved. The penetrometer was then reactivated until it reached about 40 N of contact with the firmer surface before returning to its original position.

While retracting, the force was noted to have a negative value due to the stickiness of the clay, causing the force to act in the opposite direction. When the same procedure was repeated for the second observation, a noticeable difference in the force required to reach the firmer surface was observed due to the hole formed from the first observation. Alongside the force measurements, the displacements of the penetrometer are presented in Figure 10. As shown, a distinct pattern was observed repeatedly for a particular point of observation. Furthermore, based on the observations, it was indicated that the penetrometer could consistently replicate the same observation for maximum penetration across all three observations.

A summary of the details from Figure 10 is presented in Table 1. The initial interaction between the clay and the penetrometer varied slightly based on the testing specimen’s positioning, the clay filling’s presence, and the clay surface’s flatness. As shown in Table 1, the penetrometer exhibited precise consistency when encountering the maximum contact force with harder surfaces across all observations. This value should remain constant for a specific testing point. Moreover, it demonstrated the same consistency in minimum contact force as well. The actual penetration was determined by subtracting the average probe displacement under minimum contact force from the probe displacement at initial contact. The depth of the hole created after the penetration test was measured with a Vernier caliper, along with the average measurements, are compiled in Table 1. The analysis of the penetration depth and standard deviation findings suggests that the penetrometer test yielded consistent and accurate results.

**Figure 11:**
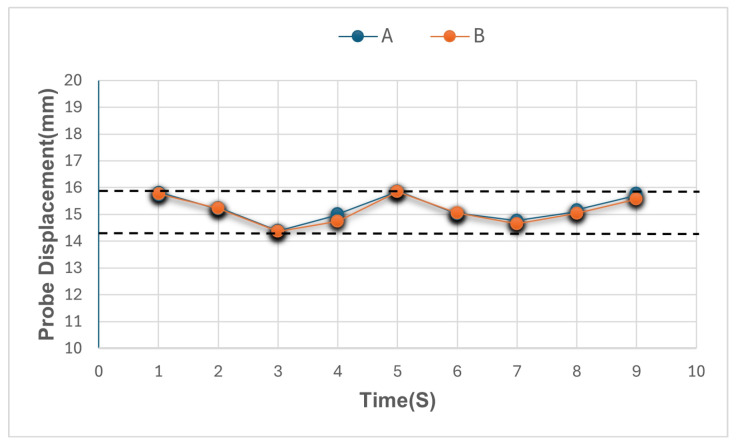
Variation in probe displacement across nine locations in the reinforced concrete sewer pipe with clay for calculated (A) and observed (B) penetration. The upper and lower limits demonstrate the variation in displacement, which is 1.49 mm, resulting from surface curvature.

Figure 11 shows some variation in actual penetration based on nine different location sites. This variation was due to the curvature of the sewer pipe, which caused fluctuations in minimum and maximum penetration across the observations. Besides the penetration through the clay, the penetrometer did not penetrate through the concrete as this concrete was not degraded.

### 4.2. Validation of Penetrometer Test with Concrete Block

**Figure 12:**
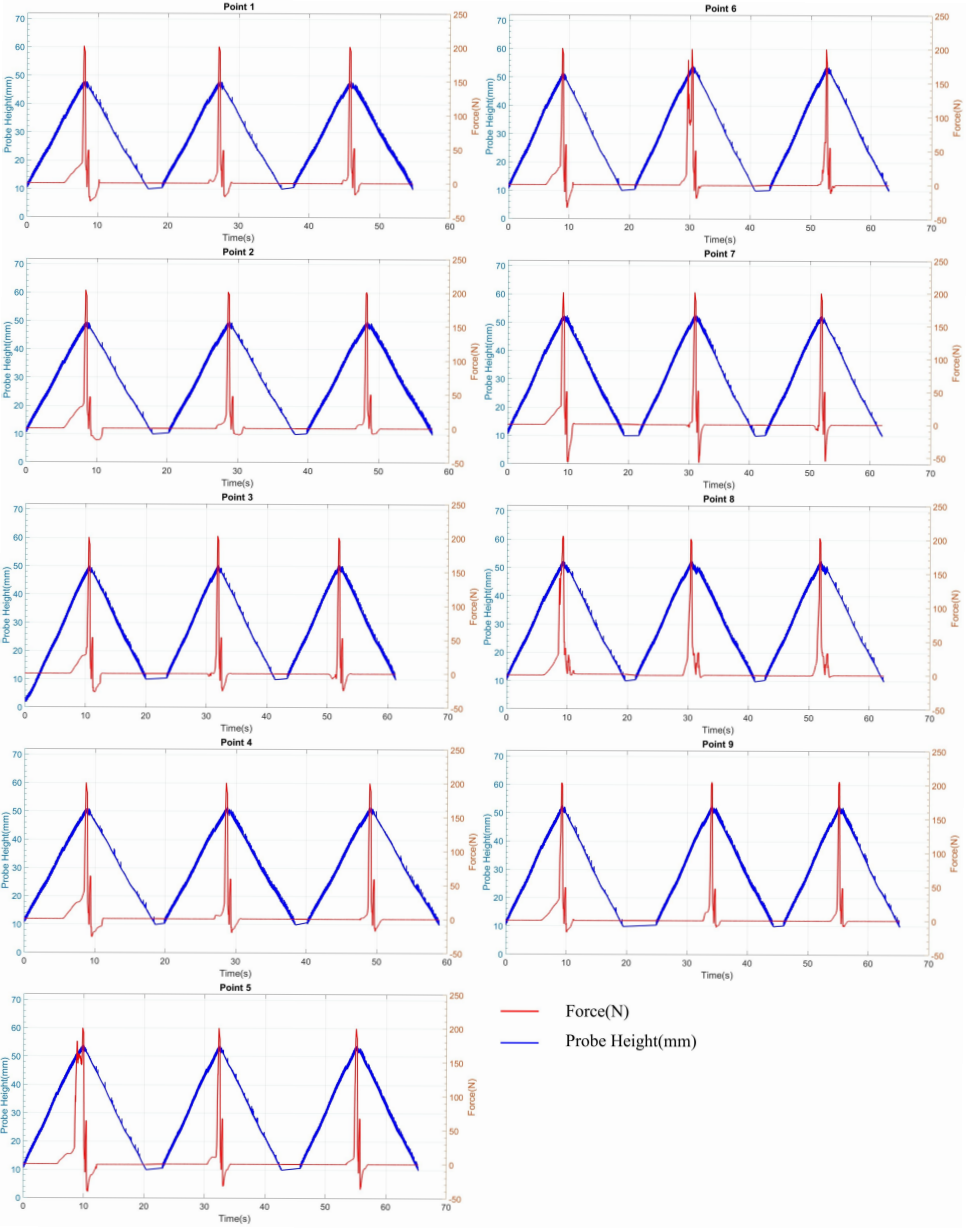
Penetrometer test with concrete block and clay for nine test sites.

For experiments involving the aggregate exposed concrete, the same (three force cycles) method was applied as for the previous tests, and similar behaviours regarding force and displacement were noted (Figure 12). However, a significant difference was observed in surface roughness; the sewer pipe featured a notably smooth surface, while the concrete block exhibited a rough surface with visible aggregates. Consequently, the penetration probe occasionally came into direct contact with the aggregates, leading to variations in both the maximum and minimum force. In Figure 12, Points 5 and 6 showed two peaks in the maximum force contact measurement. This could occur in two situations: either when the probe slightly slipped over the aggregate or when it slightly crushed the aggregate under the peak contact force of 200 N. By employing the same procedures as before, the actual penetration depth was calculated and is summarised in Table 2.

The trials indicate that the penetrometer demonstrated consistent displacement behaviour when interacting with maximum and minimum contact forces on harder surfaces across all observations. Additionally, there were minimal discrepancies between the penetrometer readings and those from the Vernier caliper (Table 2). This disparity could arise from several factors, including the shape of the measurement tool tip and potential slight damage to the exposed aggregates. However, the results indicate that the penetrometer yields more reliable readings when measuring against surfaces without exposed aggregates.

### 4.3. Sensor Deployment Mechanism

**Figure 13:**
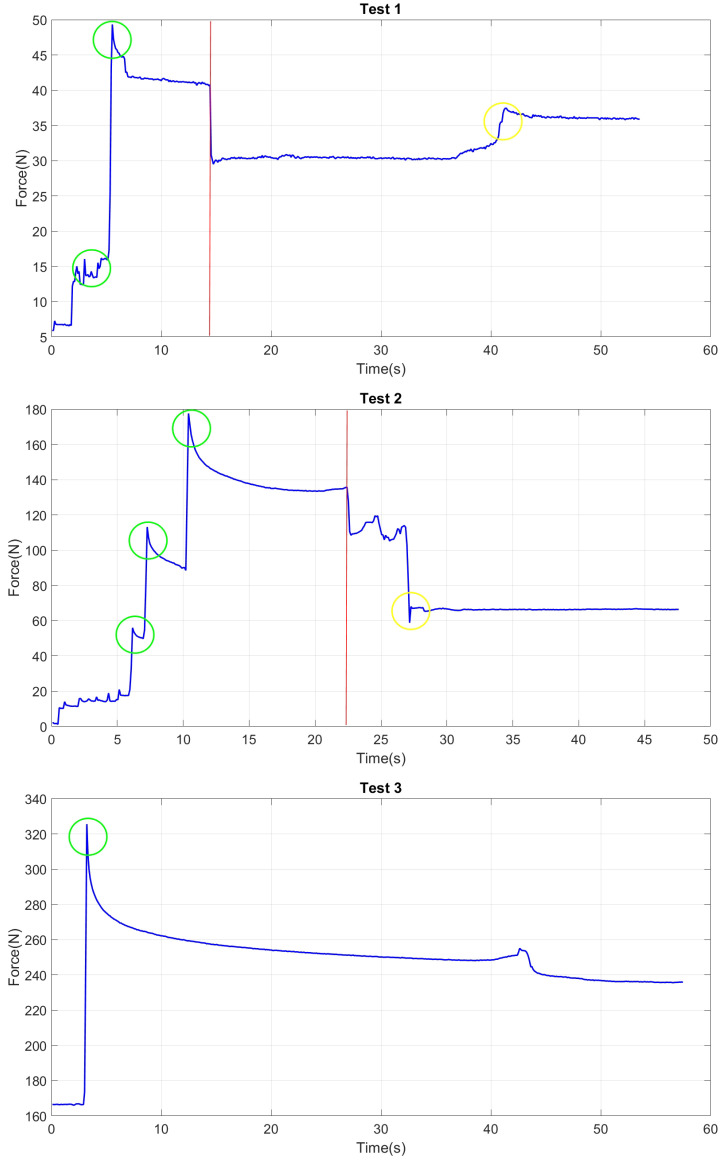
Change in contact force of the SDM under an external load on a wet wall surface. The spikes indicate the increase in contact force as the SDM’s arm is extended incrementally (green) and the stable position when the external force is no longer applied (yellow).

It is important that the SDM remains stable throughout the test procedures to allow for repeated, controlled measurements. To further investigate this point, another experimental research was conducted for three types of wall condition: dry, wet, and detergent-wet. For all three conditions, the Sensor Deployment Mechanism was adjusted to ensure contact force with the wall within three specific force ranges, from 40 N to 240 N. Figure 13 illustrates the contact force behaviour for the wet-wall study as a representative sample. Variables of the experimental study for all three types of surface, the initial contact force of the SDM, and the required external load to move the SDM are recorded respectively for each test in Table 3.

Before testing commenced, the total weight of the AMPT Robotic system was recorded as 9.8 kg. The results indicate, as would be expected, that with a lower coefficient of friction (with a wet and detergent-wet surfaces) the force required to move the robot is significantly decreased for the comparable holding force.

In the initial test, the contact force was gradually adjusted from 15 N to up to 40 N, as shown in Figure 13. Once a stable position was achieved, attempts were made to move the SDM, and the necessary weight (to shift the SDM) was recorded. The observation indicated that the minimum force required to move the SDM was approximately 15 kg. As depicted in Figure 13, once movement began some contact force with the wall was lost, clearly illustrated by the red line in Test 1. During Test 2, the contact force between the SDM and the wall was increased in increments by extending the SDM’s arms until it reached around 140 N. Once it hit the targeted range, attempts were made to move the SDM, and the force required was documented. In this instance, approximately 32 kg was needed to shift the robot. This was clearly shown in Test 2, where the SDM began to lose contact with the surface at approximately 23 s. Lastly, in Test 3, the contact force was elevated to approximately 250 N. In this scenario, a maximum force of 50 kg was applied to lift the SDM, and no movement was visibly observed. Hence, the required minimum force to move the SDM should be more than 50 kg, as shown in Table 3 in Test 3. This is also clearly illustrated in Test 3 of Figure 13. However, when testing with the detergent-wet wall, the SDM began to shift at approximately 47 kg while maintaining a contact force of around 240 N with the wall—resulting in an additional weight capacity of 37 kg for the detergent-wet wall, which is adequate for required stable testing.

## 5. Conclusions

In this study, a novel approach for conducting a penetrometer test has been introduced to tackle the issue of precision in current methods. Presently, the penetration test on sewer maintenance holes is performed manually, leading to various challenges related to risk, cost, accuracy, and thorough analysis. To address these issues, this research presents an innovative robotic design that allows for remote execution of the penetration test without the need to enter confined spaces. This approach significantly reduces the risk to the operator’s life. Furthermore, it helps to decrease the costs involved in the testing process by minimising human involvement compared to traditional methods. Additionally, the reduction of human engagement accelerates the procedure and facilitates a more comprehensive analysis effectively and efficiently. Thus, it improves the accuracy of the observations.

In this research study, comprehensive steps for designing both the penetrometer mechanism and the Sensor Deployment Mechanism were thoroughly detailed. A method to compensate for mechanical compliance was discussed, and the procedure was described in depth. Two experimental studies were conducted on both reinforced concrete sewer pipes and concrete blocks to further illustrate the precision of the readings, repeatability, and multiple observations. Besides the penetrometer mechanism, a third experimental study was performed to verify the stability of the Sensor Deployment Mechanism under external loads. The experimental results indicated that the AMPT Robot could consistently deliver readings on various surfaces, including both smooth and rough, as well as flat and curved. Furthermore, the approach employed to address the mechanical compliance issue was shown, through experimentation, to be highly effective. Consequently, the penetrometer demonstrated high consistency regarding accuracy and repeatability during angular modulated observations.

Several challenges were encountered when measuring the actual penetration using the Vernier caliper. A primary issue was the unevenness of the clay test surface after the penetrometer test. Therefore, it was necessary to ensure the surface was flat before taking measurements with the Vernier caliper for each test location. Furthermore, the direct contact between the probe and the load cell posed challenges when the probe interacted with aggregates. In those situations, the probe would bend slightly, causing issues with data collection. To effectively address these problems, improvements to the probe mechanism can be implemented to better adapt to the testing surface with more accurate alignment regarding the contact angle. Additionally, an alternative setup can be introduced to replace the direct connection between the probe and the load cell to facilitate the angle of contact. Moreover, both the design and dimensions of the penetrometer probe can be adjusted to enhance performance under various conditions.

In the stability study of the Sensor Deployment Mechanism, it has been observed that, under wet and slippery conditions on the wall, the SDM exhibits a slight drift when subjected to a load of 370 N, which is significantly more that what the robot would expect to experience in a downward direction. To facilitate safe deployment, a tripod-based rope arrangement is planned for use during testing, which will also aid in positioning and repositioning the robot. This configuration will enhance stability and provide a secure support structure, effectively addressing any potential drift.

As mentioned at the outset of this study, the penetrometer test is only one form of non-destructive testing utilised for assessing concrete. It can offer some information regarding surface hardness and can deliver measurements for observing internal diameter. In certain studies, the penetrometer test has been employed to establish empirical relationships with concrete compressive strength. Nevertheless, relying solely on a penetrometer makes it challenging to accurately evaluate the condition of the concrete due to the numerous factors affecting the structural integrity of sewer pipes. Therefore, in combination with other non-destructive testing methods, the penetrometer approach could yield more dependable insights regarding asset integrity.

## Figures and Tables

**Table 1:** Summarized penetrometer test results of reinforced concrete sewer pipe with clay.

Point No:	Initial Contact (mm)	Max. Contact (mm)	Avg. Min. Contact (mm)	Calculated Displacement (mm)	V.Calliper Measurement (mm)	Standard Deviation of Point
Point 1	20.64	36.79	36.36	15.72	15.77	0.0513
		36.79	36.43	15.79		
		36.81	36.46	15.82		
Point 2	20.62	36.21	35.81	15.19	15.22	0.0306
		36.22	35.83	15.21		
		36.22	35.87	15.25		
Point 3	20.95	35.67	35.33	14.38	14.36	0.0115
		35.67	35.35	14.40		
		35.66	35.33	14.38		
Point 4	20.77	36.10	35.75	14.98	14.74	0.0153
		36.13	35.77	15.00		
		36.14	35.78	15.01		
Point 5	20.8	37.07	36.65	15.85	15.85	0.0115
		37.07	36.65	15.85		
		37.07	36.67	15.87		
Point 6	21.35	36.78	36.36	15.01	15.05	0.0173
		36.79	36.36	15.01		
		36.79	36.39	15.04		
Point 7	21.02	36.22	35.78	14.76	14.65	0.0058
		36.21	35.78	14.76		
		36.21	35.79	14.77		
Point 8	21.11	36.64	36.21	15.10	15.04	0.0351
		36.65	36.25	15.14		
		36.67	36.28	15.17		
Point 9	20.76	36.86	36.47	15.71	15.57	0.0361
		36.87	36.52	15.76		
		36.87	36.54	15.78		

**Table 2:** Summarised penetrometer test results of concrete block with clay.

Point No:	Initial Contact (mm)	Max. Contact (mm)	Avg. Min. Contact (mm)	Calculated Displacement (mm)	V.Calliper Measurement (mm)	Standard Deviation of Point
Point 1	34.27	46.99	46.52	12.25	12.45	0.0436
		46.92	46.45	12.18		
		46.92	46.44	12.17		
Point 2	34.68	48.55	48.06	13.38	13.41	0.0971
		48.61	48.12	13.44		
		48.74	48.25	13.57		
Point 3	35.10	48.91	48.42	13.32	13.69	0.20001
		49.15	48.61	13.51		
		49.36	48.82	13.72		
Point 4	35.74	49.99	49.54	13.80	13.87	0.2554
		50.26	49.82	14.08		
		50.47	50.05	14.31		
Point 5	37.61	52.84	52.47	14.86	13.50	0.0987
		52.72	52.31	14.70		
		52.85	52.49	14.88		
Point 6	38.75	50.38	49.92	11.17	13.63	1.4723
		52.80	52.46	13.71		
		52.84	52.48	13.73		
Point 7	38.67	51.43	51.04	12.37	12.36	0.0635
		51.60	51.15	12.48		
		51.60	51.15	12.48		
Point 8	37.60	51.34	50.41	12.81	13.30	0.2778
		51.34	49.95	12.35		
		51.42	49.91	12.31		
Point 9	36.92	51.40	50.90	13.98	13.96	0.0361
		51.46	50.95	14.03		
		51.49	50.97	14.05		

**Table 3:** Summarized details of variation of contact force of SDM under the external loads.

Surface	Test 1	Test 2	Test 3
Dry	50 N/18 kg	140 N/42 kg	240 N/>50 kg
Wet	41 N/15 kg	140 N/32 kg	260 N/>50 kg
Detergent-Wet	75 N/23 kg	150 N/30 kg	240 N/47 kg

## Data Availability

The original contributions presented in this study are included in the article. Further inquiries can be directed to the corresponding author.
